# Cisplatin Protein Binding Partners and Their Relevance for Platinum Drug Sensitivity

**DOI:** 10.3390/cells9061322

**Published:** 2020-05-26

**Authors:** Sophie Möltgen, Eleonora Piumatti, Giuseppe M. Massafra, Sabine Metzger, Ulrich Jaehde, Ganna V. Kalayda

**Affiliations:** 1 Department of Clinical Pharmacy, Institute of Pharmacy, University of Bonn, 53113 Bonn, Germany; s.moeltgen@uni-bonn.de (S.M.); eleonora.piumatti@edu.unito.it (E.P.); giuseppe.massafra@edu.unito.it (G.M.M.); u.jaehde@uni-bonn.de (U.J.); 2 Cologne Biocenter, MS Facility, University of Cologne, 50923 Cologne, Germany; s.metzger@uni-koeln.de; 3 Leibniz Research Institute for Environmental Medicine, 40225 Düsseldorf, Germany; 4 Federal Institute for Drugs and Medical Devices (BfArM), 53175 Bonn, Germany

**Keywords:** cisplatin resistance, BODIPY-cisplatin, oxaliplatin, vimentin, glutathione-S-transferase π, growth factor receptor-bound protein 2, FiVe1

## Abstract

Cisplatin is a widely used drug in the treatment of various solid tumors, such as ovarian cancer. However, while the acquired resistance significantly limits the success of therapy, some tumors, such as colorectal cancer, are intrinsically insensitive to cisplatin. Only a small amount of intracellular platinum binds to the target—genomic DNA. The fate of the remaining drug is largely obscure. This work aimed to identify the cytosolic protein binding partners of cisplatin in ovarian and colorectal cancer cells and to evaluate their relevance for cell sensitivity to cisplatin and oxaliplatin. Using the fluorescent cisplatin analog BODIPY-cisplatin, two-dimensional gel electrophoresis, and mass spectrometry, we identified the protein binding partners in A2780 and cisplatin-resistant A2780cis ovarian carcinoma, as well as in HCT-8 and oxaliplatin-resistant HCT-8ox colorectal cell lines. Vimentin, only identified in ovarian cancer cells; growth factor receptor-bound protein 2, only identified in colorectal cancer cells; and glutathione-S-transferase π, identified in all four cell lines, were further investigated. The effect of pharmacological inhibition and siRNA-mediated knockdown on cytotoxicity was studied to assess the relevance of these binding partners. The silencing of glutathione-S-transferase π significantly sensitized intrinsically resistant HCT-8 and HCT-8ox cells to cisplatin, suggesting a possible involvement of the protein in the resistance of colorectal cancer cells to the drug. The inhibition of vimentin with FiVe1 resulted in a significant sensitization of A2780 and A2780cis cells to cisplatin, revealing new possibilities for improving the chemosensitivity of ovarian cancer cells.

## 1. Introduction

Even in times of targeted- and immunotherapies, traditional chemotherapy, such as platinum drugs, remains a backbone in the treatment of various solid tumors [1]. The major drawback of platinum-based chemotherapy is inherent and acquired resistance [2,3]. Studies have shown that only small amounts of intracellular platinum reach the nucleus and can thus interact with DNA, leading to apoptosis [4]. Mechanisms that result in a less effective influx or more pronounced efflux of the drug and contribute to resistance have been thoroughly investigated [2,3,5]. On the contrary, the fate of cisplatin in the cytosol and the relevance of alternative binding partners for tumor cell sensitivity and resistance have not yet been fully elucidated.

Cisplatin is the oldest platinum drug approved, and while it is very effective in many tumor entities, its use is limited by severe side effects, such as nephropathy and emesis, often leading to the termination of treatment [1,6]. Therefore, the development of alternative, just as efficient, platinum drugs, with more favorable adverse effect profiles, such as the second-generation platinum drug carboplatin and third-generation platinum drug oxaliplatin, has been considered a monumental achievement [2]. Interestingly, oxaliplatin differs from its predecessors in terms of its unique spectrum of activity and distinctive side effects [7,8]. The exact mechanism of action of oxaliplatin in comparison to the other two platinum drugs has not yet been resolved in detail [8,9]. In this respect, a comparison of drug action in colorectal and ovarian cancer is of particular interest, since cisplatin is used in ovarian cancer treatment with good primary response rates, whereas colorectal cancer is intrinsically resistant to this drug [10]. Remarkably, there seems to be no cross-resistance to oxaliplatin [11], which is the first-line treatment for advanced colorectal cancer [12].

The development of resistance is a phenomenon that not only leads to the absence of a positive risk–benefit ratio and dominance of unwanted adverse reactions, but also hinders total eradication of the tumor in many cases and subsequently results in increasing mortality rates [13,14,15]. Therefore, it is of the utmost importance to unravel unknown details about resistance mechanisms, and in doing so, identify biomarkers allowing the early identification of resistant tumors. On the other hand, the characterization of alternative drug targets may help to expand the possibilities of cancer therapies through the development of effective combination treatments. In our previous work, we successfully identified cytosolic binding partners of CFDA-cisplatin (cisplatin analog featuring a carboxyfluorescein diacetate tag) in ovarian cancer cells [16,17]. We showed that the pharmacological inhibition of protein disulfide isomerase A1 (PDIA1) resulted in a restored sensitivity of resistant cells to cisplatin [18]. A similar attempt was conducted by Karasawa et al., who synthesized platinum-agarose conjugates to specifically investigate protein binding that might be involved in two common cisplatin side effects—ototoxicity and nephrotoxicity [19]. Furthermore, Messori et al. emphasized that the exploration of cisplatin–protein interactions is essential for studying resistance mechanisms and the development of new therapeutic agents. Therefore, they made an effort to elucidate the critical characteristics of cisplatin binding to proteins and pointed out that this would eventually help to predict possible binding partners [20].

Given the different activity of cisplatin in ovarian and colorectal cancer, we aimed to identify cytosolic protein binding partners of cisplatin in tumor cells of these two entities and evaluate the relevance of selected proteins for cytotoxicity in sensitive and drug-resistant cells. For the latter purpose, we used specific inhibitors and silencing of genes coding for the respective proteins.

## 2. Materials and Methods

### 2.1. Reagents

RPMI 1640 cell culture media and trypsin-EDTA were obtained from Thermo Fisher Scientific (Rockford, Illinois, IL, USA). Cis-diamminedichloridoplatinum (II) (cisplatin), ammonium persulfate, glycerine, dithiothreitol (DTT), acetonitrile, ammonium bicarbonate, acrylamide, formic acid, 2-mercaptoethanol, bovine serum albumin (BSA), pepstatin, leupeptin, sodium orthovanadate, and protein inhibitor cocktail were purchased from Sigma-Aldrich (Steinheim, Germany). Fetal bovine serum, penicillin/streptomycin, and Phosphate Buffered Salt Solution (PBS) were obtained from Pan Biotech (Aidenbach, Germany). Urea, 3-[(3-Cholamidopropyl) dimethylammonio]-1-propanesulfonate (CHAPS) and Laemmli Buffer were obtained from Serva Electrophoresis (Heidelberg, Germany). 3-(4,5-Dimethylthiazol-2-yl)-2,5-diphenyltetrazolium bromide (MTT), thiourea, DeStreak solution (HED), DryStrip cover fluid, *N*,*N*,*N*’,*N*’-tetramethylethylenediamine (TEMED), tris(hydroxymethyl)aminomethane, iodoacetamide (IAA), glycine, and Tween-20 were received from VWR International (Darmstadt, Germany). Bromophenol blue and sodium fluoride were acquired from Applichem (Darmstadt, Germany). Rotiphorese^®^ Gel 30 and sodium dodecyl sulfate (SDS) were purchased from Roth (Karlsruhe, Germany). Sequencing grade trypsin was obtained from Promega (Mannheim, Germany), sodium azide was obtained from Merck Schuchardt OHG (Hohenbrunn, Germany), sodium chloride was obtained from Fisher Scientific (Hampton, Commonwealth of Virginia, VA, USA), and skim milk powder was obtained from LABC-Labortechnik (Hennef, Germany). Carboxyl-BODIPY was ordered from Lumiprobe (Hannover, Germany). The inhibitors FiVe1 and Ezatiostat-HCl were purchased from Biotrend (Cologne, Germany). The Grb2 inhibitors A and B were obtained from NCI/DTP Open Chemical Repository. BODIPY-cisplatin was prepared according to the literature procedure [21].

### 2.2. Cell Culture

The human ovarian carcinoma cell line A2780 (catalog nr. 93112519) and its cisplatin-resistant subline A2780cis (catalog nr. 93112517) were acquired from the European Collection of Authenticated Cell Cultures (ECACC), UK. The human ileocecal colorectal adenocarcinoma cell line HCT-8 and its oxaliplatin-resistant sub-cell line HCT-8ox were kindly provided by Dr. R.A. Hilger, University of Essen, Germany. All cell lines were cultured in RMPI 1640 medium at 37 °C and 5% CO_2_. The culture media were supplemented with 10% fetal bovine serum, 100 I.E./mL penicillin, and 0.1 mg/mL streptomycin.

### 2.3. Two-Dimensional Gel Electrophoresis and the Detection of Binding Partners

Two-dimensional gel electrophoresis and all associated procedures (such as precipitation, isoelectric focusing, and staining) were carried out according to Kotz et al. [16], with slight modifications to the protocol. Cells were grown in T175 cell culture flasks until 90% confluence and then treated with 25 µM of BODIPY-cisplatin for 2 h. After several washing steps and fractionation according to the instructions of the Nuclear/Cytosol Fractionation Kit (BioVision, Milpitas, California, CA, USA), cytosolic fractions were subjected to 2D gel electrophoresis. All experiments were conducted with the PROTEAN IEF Cell (Bio-Rad Laboratories, Munich, Germany) in the first dimension, followed by the second-dimension SDS-PAGE. After precipitation according to Wessel et al. [22], 150 µg of proteins (as determined by the Pierce™ BCA Protein Assay Kit (Thermo Fisher Scientific, Rockford, Illinois, IL, USA), according to the manufacturer’s instructions) was solubilized in 7 M urea, 2 M thiourea, 2% CHAPS, 12 µL HED, and 0.5% SERVALYT™ 3–10 (Serva, Heidelberg, Germany) for 1 h. Subsequently, in-gel rehydration of Serva IPG BlueStrips pH 3–10 NL (70 × 3 × 0.5 mm; Serva, Heidelberg, Germany) was performed overnight. After approximately 24 h, isoelectric focusing was carried out with a maximum current of 50 µA and a total of 17–18 kVh (300 V for 12 h (rapid), 1000 V for 0.5 h (linear), 3000 V for 1.5 h (linear), and 3000 V for 3.5 h (rapid)). All focused strips were stored at −80 °C until equilibration. SDS-PAGE was preceded by reduction and alkylation by 1% DTT and 2.5% IAA in 10 mL equilibration solution (6 M urea, 50 mM Tris-HCl (pH 8.8), 30% glycerol, and 4% SDS) per strip. Afterwards, the strip was placed on a ready-made 8–16% separation gel (Serva, Heidelberg, Germany) flanked by two electrode wicks soaked with protein marker (PageRuler™ Plus Prestained Protein Ladder; Thermo Fisher Scientific, Rockford, Illinois, IL, USA). It was then sealed with a 4% stacking gel to prevent the strip from moving and to ensure exact and air bubble-free contact of the IPG strip and separation gel. Proteins were separated for approximately 1.5 h, starting at 80 V for 0.5 h, followed by 180 V, until the running front reached the bottom of the gel.

After the gels were scanned using the ChemiDoc™ MP Imaging System (Bio-Rad Laboratories, Munich, Germany) to detect fluorescence at 488/532 nm (excitation/emission), they were stained with Coomassie (Quick Coomassie^®^ Stain, Serva, Heidelberg, Germany) overnight to visualize all proteins. On the next day, the gels were scanned again, and Delta2D (version 4.8; Decodon, Greifswald, Germany) was used to warp the fluorescence and Coomassie-stained pictures. The experiment was reproduced three times using the cytosolic fraction of different cell passages. Spots, where both BODIPY-cisplatin and protein staining were detected, were cut out and prepared for mass spectrometry.

### 2.4. Mass Spectrometry

#### 2.4.1. Sample Preparation for MS

For protein identification, gel slices were subjected to in-gel digestion [23,24]. In brief, slices were washed consecutively with water, 50% acetonitrile (ACN), and 100% ACN. Proteins were reduced with 20 mM DTT in 50 mM ammonium bicarbonate and alkylated with 40 mM acrylamide (in 50 mM bicarbonate) for 30 min. The slices were rewashed and dehydrated with acetonitrile. Dried slices were incubated with 330 ng trypsin at 37 °C overnight. The peptide extract was separated, and remaining peptides were extracted with 50% acetonitrile. Peptides were dried in a vacuum concentrator and stored at −20 °C.

#### 2.4.2. Protein Identification by MS

Peptides were dissolved in 0.1% formic acid (solvent A) and 1/3 was injected into a C18 trap column (20 mm length, 100 µm inner diameter, ReproSil-Pur 120 C18-AQ, 5 µm, Dr. Maisch GmbH, Ammerbuch-Entringen, Germany) made in-house. Bound peptides were eluted on a C18 analytical column (200 mm length, 75 µm inner diameter, ReproSil-Pur 120 C18-AQ, 3 µm). Peptides were separated during a linear gradient from 2% to 35% solvent B (90% acetonitrile, 0.1% formic acid) within 20 min at 300 nL/min. The nanoHPLC was coupled online to an LTQ Orbitrap Velos mass spectrometer (Thermo Fisher Scientific, Bremen, Germany). Peptide ions between 330 and 1600 *m*/*z* were scanned in the Orbitrap detector with a resolution of 30,000 (maximum fill time of 400 ms, AGC target of 10^6^). The 20 most intense precursor ions (threshold intensity of 3000, isolation width of 1.1 Da) were subjected to collision-induced dissociation (normalized energy of 35) and analyzed in the linear ion trap. Fragmented peptide ions were excluded from repeat analysis for 13 s.

Raw data processing and an analysis of database searches were performed with Proteome Discoverer software 2.2.0.388 (Thermo Fisher Scientific, Commonwealth of Massachusetts, MA, USA). Peptide identification was completed with an in-house Mascot server version 2.6.1 (Matrix Science Ltd., London, UK). MS2 data were searched against human sequences in SwissProt (release 2018_10) and common contaminants. The mass-to-charge ratio tolerance was 10 ppm (precursor ions) and 0.6 Da (fragment ions), respectively. Tryptic peptides with up to two missed cleavages were searched. Propionamide, PtBDP (BC_17_F_2_H_26_N_5_O_2_Pt mass shifts of 576.178992 and 557.160602 with and without water, respectively) were set as dynamic modifications on cysteines. PtBDP modifications as above, but without one hydrogen, were dynamically searched on cysteine, histidine, and methionine. The oxidation of methionine and *N*-terminal protein acetylation were also allowed as dynamic modifications. Mascot results were assigned q-values by the percolator algorithm [25] version 3.00, as implemented in Proteome Discoverer. The localization of modifications was scored with the ptmRS 2.0 node [26]. Proteins were included if at least two peptides were identified with q ≤ 0.01. Actual false positive rates were typically ≈1% on PSM, peptide, and protein levels. 

### 2.5. Small-Interfering RNA-Mediated Knockdown

Cells were seeded in 6-well plates at a density of 5 × 10^5^ cells/well for A2780 cells and 2.5 × 10^5^ cells/well for HCT-8 cells, and were allowed to attach overnight at 37 °C and 5% CO_2_. On the next day, cells were treated with 100 pmol protein-specific siRNA for GSTP1 (Thermo Fisher Scientific, Rockford, Illinois, IL, USA; HSS104546), vimentin (Thermo Fisher Scientific, Rockford, Illinois, IL, USA; s14799), Grb2 (Thermo Fisher Scientific, Rockford, Illinois, IL, USA; s226232), or negative control siRNA (Thermo Fisher Scientific, Rockford, Illinois, IL, USA; 12935112) and the K4^®^ Transfection System (Biontex Laboratories, Munich, Germany), according to the manufacturer’s instructions. First, cells were pre-treated with 10 µL K4^®^ multiplier per well. For two wells of a 6-well plate, 10 µL of siRNA stock (20 µM) was mixed with 250 µL medium, without serum and antibiotics. In another tube, 27 µL of K4^®^ transfection reagent was mixed with 250 µL of medium. Then, 260 µL of the transfection reagent dilution was added to 260 µL of the siRNA dilution and incubated at room temperature for 15 min. A total of 250 µL of the mixture per well was added to the cells, and the plate was gently swayed to ensure an equal distribution of the transfection complex. After 24 h, siRNA-containing antibiotic-free medium was removed and replaced by fresh full medium. After another 48 h, cells were lysed with lysis buffer (RIPA buffer pH 7.4, Pepstatin A, Leupeptin, NaF, Na_3_VO_4_, protease inhibitor cocktail) and sonicated (50% power, 5–30 sec pause, 3×) to ensure protein solubilization. Next, lysates were centrifuged, and the supernatant was stored at −80 °C until further use.

### 2.6. Cytotoxicity Assay

Cells were seeded in 96-well plates at a density of 3 × 10^3^ cells/well for HCT-8 cells and 1 × 10^4^ cells/well for A2780 cells, and allowed to attach overnight at 37 °C and 5% CO_2_. The following day, the cells were exposed to different concentrations of substances (either the platinum drug alone, inhibitor alone, or a combination of both) for 72 h, unless otherwise specified. After that, cells were treated with MTT for 1 h. The supernatant was subsequently removed, and the purple formazan crystals produced by viable cells were dissolved in DMSO. The absorbance was quantified at 570 nm with background subtraction at 690 nm. Cytotoxicity experiments after knockdown followed a slightly different procedure: after seeding of the cells and overnight incubation, they were treated with siRNA (8 pmol/well) for 24 h, as described above, and then exposed to the platinum drug for 48 h. The pEC_50_ and EC_50_ were determined using non-linear regression analysis with GraphPad Prism^®^ 6.0 (sigmoidal dose-response, variable slope). The resistance factor was calculated by dividing the EC_50_ of resistant cells by the EC_50_ of sensitive cells.

### 2.7. Apoptosis Assay

Apoptosis was assessed using the eBioscience™ Annexin V-FITC Apoptosis Detection Kit (Thermo Fisher, Rockford, Illinois, IL, USA), according to the manufacturer’s instructions. Cells were seeded in 6-well plates at a density of 1 × 10^5^ cells/well. After having attached overnight at 37 °C and 5% CO_2_, they were exposed to either platinum drug/inhibitor combinations for 72 h or siRNA for 24 h, followed by platinum drug exposure for 48 h. The treated cells were then incubated with propidium iodide (PI) and Annexin V-FITC for 15 min at room temperature in the dark and measured by flow cytometry (Guava^®^ easyCyte™ HT, Luminex Corporation, Austin, Texas, TX, USA). Subsequent analysis was conducted with Guava^®^ InCyte™ (version 3.3; Luminex Corporation, Austin, Texas, TX, USA). Annexin V-FITC negative/PI negative cells were considered to be alive. Annexin V-FITC positive/PI negative cells were considered to be early apoptotic. Annexin V-FITC negative/PI positive and Annexin V-FITC positive/PI positive cells were combined and considered to be late apoptotic/necrotic. Cellular debris was excluded using forward and side scatter.

### 2.8. Combination Index

In order to determine the combination index for cisplatin and FiVe1, experiments were performed and analyzed with the CompuSyn^®^ software, according to the method by Chou et al. [27]. A2780 cells were seeded in 96-well plates at a density of 1 × 10^4^ cells/well and incubated overnight at 37 °C and 5% CO_2_. The following day, they were treated with either 0–100 µM cisplatin or FiVe1 alone, or with combinations of 10%, 20%, 40%, 60%, 80%, 100%, 200%, 400%, and 800% of the previously determined EC_50_ concentrations of cisplatin and FiVe1. The ratio of cisplatin to FiVe1 in A2780 was 1.247 and in A2780cis, it was 6.225. After 72 h, the modalities of the combinations were assessed via an MTT-based assay. As specified by Chou et al., values > 1, =1, and <1 correspond to antagonism, additivity, and synergism, respectively [27].

### 2.9. Western Blot Analysis

After the siRNA-mediated knockdown and lysis of protein samples, the total protein amount was determined by the Pierce™ BCA Protein Assay Kit (Thermo Fisher Scientific, Rockford, Illinois, IL, USA), according to the manufacturer’s instructions. A 20 µg total protein/gel pocket was loaded on 12% gels, separated using SDS-PAGE, and then transferred to a PVDF membrane (100 V, 350 mA, 2 h). After blocking the membrane in 5% skim milk in tris-buffered saline combined with 0.2% Tween 20 (TBS-T) for 1 h, it was washed several times, before being subjected to the primary antibody against GSTP1 (Santa Cruz Biotechnology, Dallas, Texas, TX, USA; sc-66000), vimentin (Santa Cruz Biotechnology, Dallas, Texas, TX, USA; sc-6260), or Grb2 (Santa Cruz Biotechnology, Dallas, Texas, TX, USA; sc-8034) overnight at 4 °C. On the next day, the membrane was washed again and then treated with the secondary antibody (goat anti-rabbit, 4030-05 or goat anti-mouse, 1030-05, both Southern Biotech, Birmingham, AL, USA) for 1.5 h. The proteins were visualized with the Pierce™ ECL Western Blotting Substrate Kit (Thermo Fisher Scientific, Rockford, Illinois, IL, USA) on a ChemiDoc™ XRS+ System (Bio-Rad Laboratories, Munich, Germany) and densitometrically evaluated with ImageLab software (version 5.1; Bio-Rad Laboratories, Munich, Germany). GAPDH (GeneTex, Irivine, California, CA, USA; GTX100118) expression was used as a loading control. 

### 2.10. Statistical Analysis

Statistical comparisons between groups were carried out using either an unpaired *t*-test (in the case of cytotoxicity experiments) or a one-way analysis of variance (ANOVA) with a Sidak post-hoc test (for all other experiments) using GraphPad Prism^®^ 6.0. Differences were considered statistically significant for *p* < 0.05. 

## 3. Results

### 3.1. Fluorescent Cisplatin Analog BODIPY-Cisplatin

**Figure 1:**
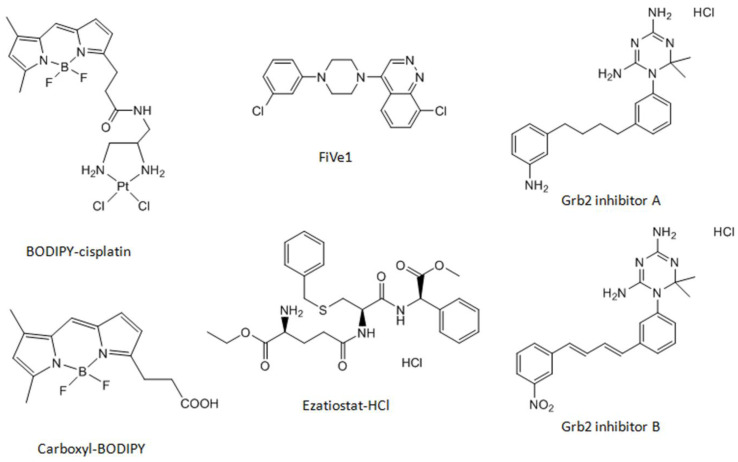
Chemical structures of the cisplatin analog BODIPY-cisplatin, platinum-free label carboxyl-BODIPY, and the inhibitors used in this study.

We chose a fluorescent cisplatin analog tagged with boron-dipyrromethene (BODIPY-cisplatin, Figure 1, [21]) to detect potential protein binding partners of cisplatin. We could show that BODIPY-cisplatin acts similarly to its parent drug with regard to cytotoxicity, even though it was reduced due to the introduction of the tag (Table 1). Cisplatin-resistant A2780cis ovarian cancer cells exhibited resistance towards BODIPY-cisplatin (resistance factor (RF) was 4.2 for cisplatin and 5.4 for BODIPY-cisplatin). The cytotoxicity of BODIPY-cisplatin was much lower in colorectal cancer cells, both in the oxaliplatin-sensitive HCT-8 and oxaliplatin-resistant HCT-8ox cell lines, which are intrinsically resistant to cisplatin. In HCT-8 cells, cisplatin was 4.7 times less active than in A2780 ovarian cancer cells. The cytotoxicity of BODIPY-cisplatin was reduced 5.2-fold. The platinum-free label carboxyl-BODIPY (Figure 1) showed no antitumor activity in all four cell lines up to 500 µM. It should be noted that cisplatin-resistant A2780cis cells exhibited some degree of cross-resistance to oxaliplatin (ca. 3-fold), and vice versa, oxaliplatin-resistant HCT-8ox cells were somewhat cross-resistant to cisplatin (less than 2-fold). 

### 3.2. Detection and Identification of Binding Partners of BODIPY-Cisplatin

**Figure 2:**
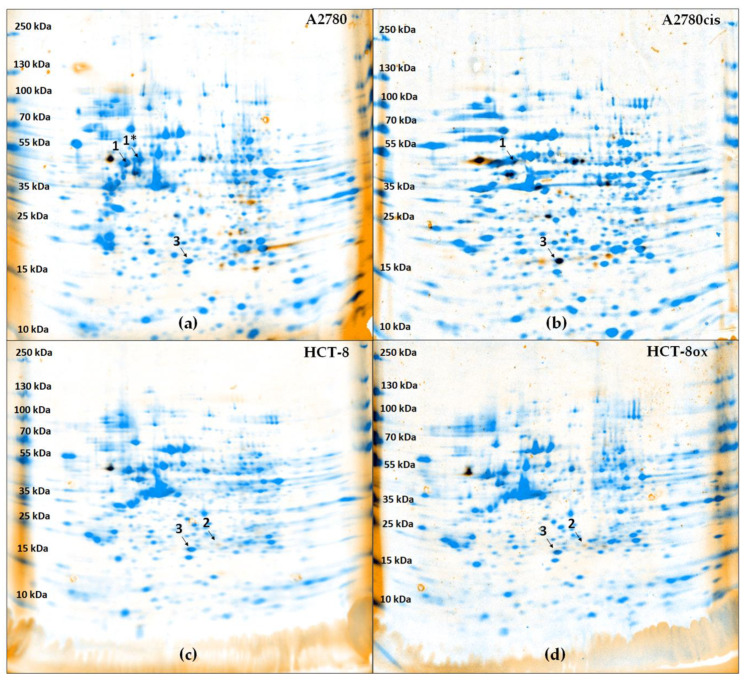
Overlay of fluorescence image and protein staining (Coomassie) in gels after two-dimensional electrophoresis (pH 3–10 NL) of each 20 µg cytosolic fraction of (**a**) A2780, (**b**) A2780cis, (**c**) HCT-8, and (**d**) HCT-8ox cells treated with BODIPY-cisplatin. Spots 1 and 1* were identified as vimentin, spot 2 as Grb2, and spot 3 as GSTP1.

After the separation of cytosolic proteins via two-dimensional gel electrophoresis, the combination of the fluorescence detection of BODIPY-cisplatin and colloidal Coomassie staining of proteins led to the visualization of spots representing cytosolic cisplatin protein binding partners (Figure 2, Figure S1). We were able to show that their number differentiated between wildtype and resistant cells, on the one hand (Figure S1 a vs. b and c vs. d), and between cells with intrinsic and acquired cisplatin resistance (Figure S1 b vs. c and b vs. d), on the other hand. While there were many fluorescent spots in both A2780 and A2780cis cells, BODIPY-cisplatin signals did not overlay to the same extent with the protein staining of HCT-8 and HCT-8ox cells. Some of the cytosolic proteins identified (Table S1) in A2780 and A2780cis cells were the same as previously reported and studied in detail, such as protein disulfide isomerase 1 (PDIA1) [16,17,18]. For further investigation, we selected one protein only identified in ovarian cancer cells (vimentin, Figure 2, spot 1, 1*), one protein only identified in colorectal cancer cells (growth receptor factor bound protein 2, Grb2, Figure 2, spot 2), and one protein identified in all four cell lines (glutathione-S-transferase π, GSTP1, Figure 2, spot 3). These proteins were chosen due to the great evidence of their relevance for cancer drug resistance [28,29,30]. Other binding partners will be investigated in our further studies. 

### 3.3. Effect of the Pharmacological Inhibition and Knockdown of Vimentin on Cisplatin Sensitivity

**Figure 3:**
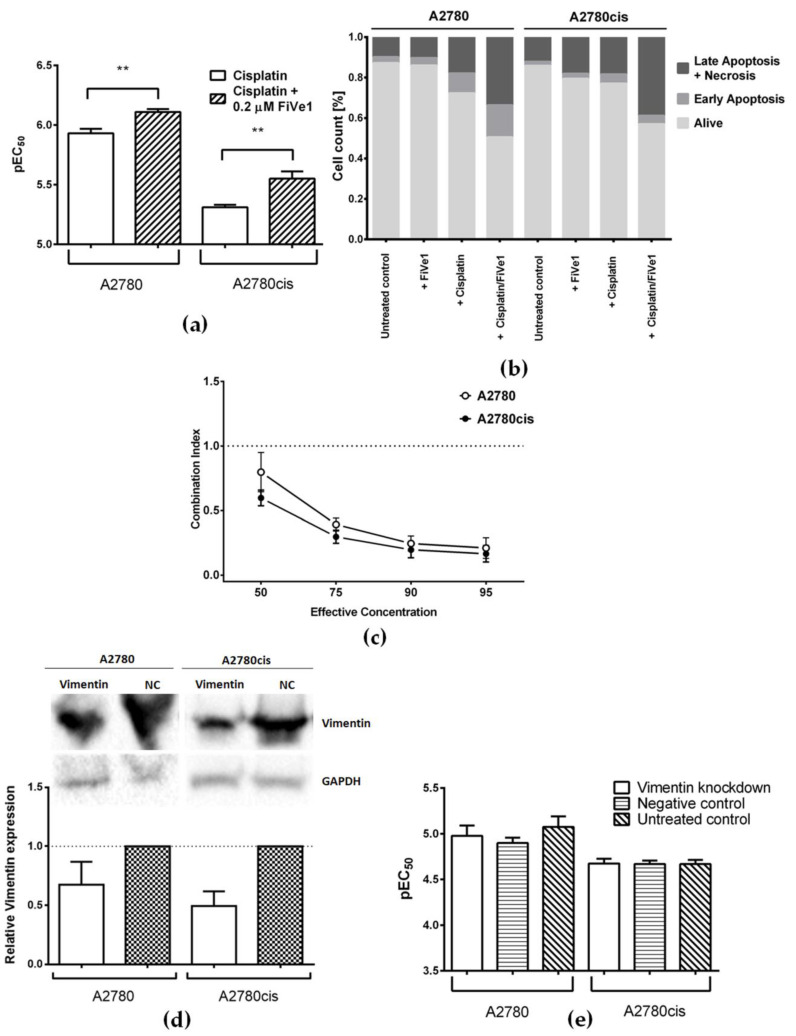
(**a**) Cisplatin cytotoxicity in A2780 and A2780cis cells alone or upon co-incubation with 0.2 µM FiVe1 (mean ± SEM, *n* = 4–6); (**b**) percentage of early apoptotic, late apoptotic, and necrotic, as well as alive, cells in A2780 and A2780cis cells after the co-incubation of cisplatin with FiVe1, in comparison to the treatment with each of the compounds alone and untreated cells; (**c**) Combination Index (CI) of cisplatin and FiVe1; as described by Chou et al. [27], CI was determined at effective concentrations from EC_50_ to EC_95_ (mean ± SEM, *n* = 8); (**d**) representative Western Blots and densitometric quantification of protein expression after treatment with vimentin-specific and negative control (NC) siRNA in A2780 and A2780cis cells, where GAPDH served as a loading control (mean ± SEM, *n* = 3); (**e**) cisplatin cytotoxicity in A2780 and A2780cis cells after vimentin knockdown, prior treatment with negative control siRNA, or no pre-treatment (mean ± SEM, *n* = 4). ** *p* < 0.01.

Vimentin was identified as a BODIPY-cisplatin binding partner in both ovarian cancer cell lines (Figure 2, spot 1 and 1*). In order to assess the relevance of vimentin for cisplatin cytotoxicity, we used the recently developed vimentin inhibitor FiVe1 (Figure 1, [31]). After determining the non-toxic FiVe1 concentration (EC_50_ was 0.93 µM in A2780 and 0.80 µM in A2780cis), cells were subjected to cisplatin in combination with 0.2 µM FiVe1 over 72 h. Both A2780 and A2780cis cells were significantly sensitized towards cisplatin (in A2780 EC_50_, the value changed from 1.17 to 0.78 µM, and in A2780cis, from 4.88 to 2.81 µM, Figure 3a). The resistance factor of A2780cis cells was reduced from 4.2 to 3.6.

Furthermore, a combination of cisplatin with 0.2 µM FiVe1 induced more pronounced apoptosis than treatment with cisplatin alone. The percentage of late apoptotic cells increased by 15.8% (*p* = 0.0071) in A2780 and by 20.4% (*p* < 0.0001) in A2780cis. The inhibitor of vimentin was used in the non-toxic concentration as mentioned above, and showed no signs of increased apoptosis when applied alone (Figure 3b and Figure S2a).

In order to understand the pharmacological interaction between cisplatin and FiVe1, the combination index was determined. It is especially interesting that the drug combination worked synergistically at effective concentrations of EC_50_ and higher, with a more pronounced effect in the resistant cell line (Figure 3c). 

In addition to pharmacological inhibition, we studied the effect of gene silencing on cell sensitivity. A typical knockdown experiment started with siRNA-mediated transfection for 24 h, followed by exposure to a platinum drug for 48 h. The expression of vimentin as detected by Western Blot 48 h after transfection was decreased only by 33% and 51% in A2780 and A2780cis, respectively, when compared to cells transfected with negative control siRNA (Figure 3d). Accordingly, no changes in cisplatin cytotoxicity after vimentin knockdown could be detected (Figure 3e).

### 3.4. Effect of the Pharmacological Inhibition and Knockdown of Grb2 on Platinum Drug Sensitivity

**Figure 4:**
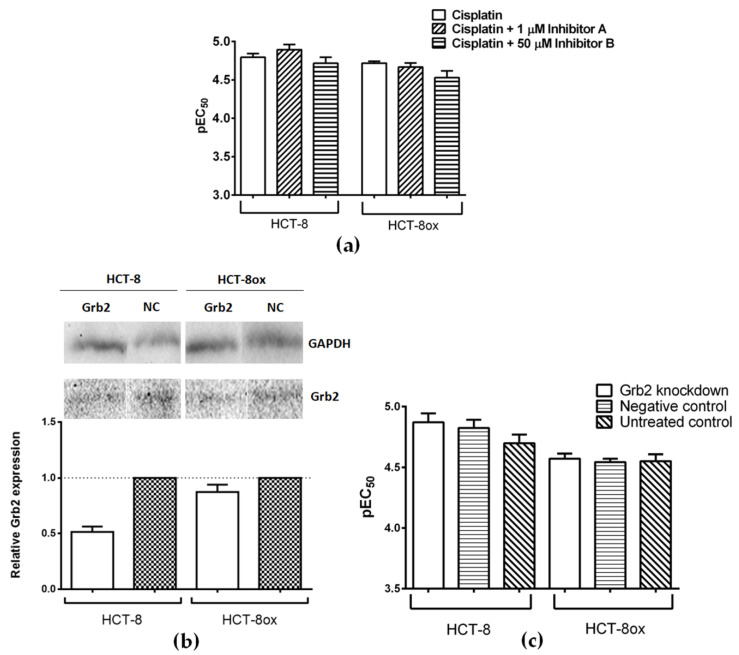
(**a**) Cisplatin cytotoxicity in HCT-8 and HCT-8ox cells alone or upon co-incubation with either 1 µM inhibitor A or 50 µM inhibitor B (mean ± SEM, *n* = 5–6); (**b**) representative Western Blots and densitometric quantification of protein expression after treatment with Grb2-specific and negative control (NC) siRNA in HCT-8 and HCT-8ox cells, where GAPDH served as a loading control (mean ± SEM, *n* = 3); (**c**) cisplatin cytotoxicity in HCT-8 and HCT-8ox cells after Grb2 knockdown, prior treatment with negative control siRNA, or no pre-treatment (mean ± SEM, *n* = 3).

Grb2 was only identified in colorectal cancer cells (Figure 2, spot 2). Since oxaliplatin is the platinum drug of choice in colorectal cancer treatment, all experiments including HCT-8 and HCT-8ox cells were carried out with oxaliplatin, in additional to cisplatin. Two inhibitors of Grb2 were evaluated—inhibitor A and inhibitor B—both developed by Simister at al. (Figure 1, [32]). Non-toxic concentrations for further experiments were selected based on prior cytotoxicity testing of the inhibitors alone: 1 µM for inhibitor A (EC_50_: HCT-8, 9.15 µM; HCT-8ox, 2.88 µM) and 50 µM for inhibitor B (EC_50_: HCT-8, 169.60 µM; HCT-8ox, 86.66 µM). There were no significant alterations in cell sensitivity to cisplatin upon co-incubation with either of the two inhibitors (Figure 4a). In terms of oxaliplatin, inhibitor A did not affect the cell sensitivity to the drug, whereas the combination with inhibitor B resulted in a reduction in sensitivity in HCT-8 cells (EC_50_ increased from 2.17 to 5.43 µM, *p* = 0.0061), but not in the oxaliplatin-resistant HCT-8ox cell line (Figure S3a). 

After the knockdown, the amount of Grb2 decreased by 48% in HCT-8 and by 13% in HCT-8ox cells (Figure 4b). This was not sufficient to affect the platinum drug sensitivity in both cell lines (Figure 4c and Figure S3b, respectively).

### 3.5. Effect of the Pharmacological Inhibition and Knockdown of GSTP1 on Platinum Drug Sensitivity

**Figure 5:**
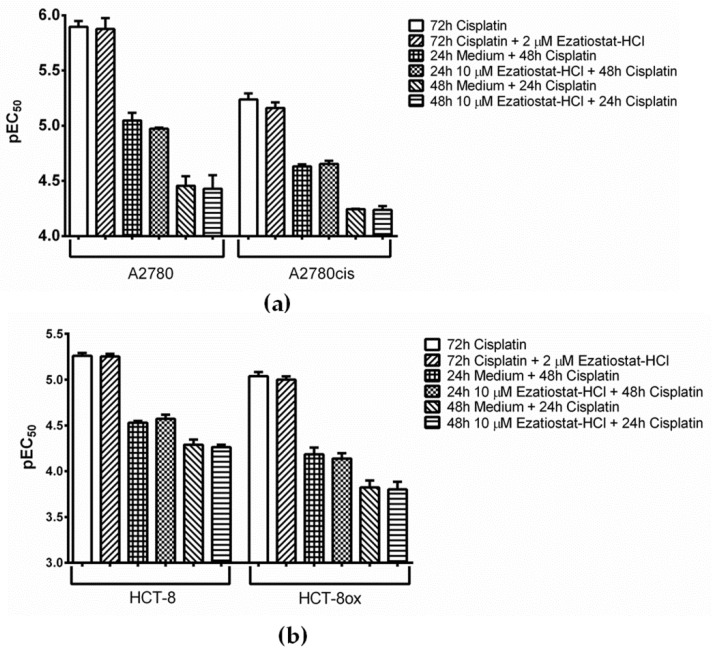
Cisplatin cytotoxicity (**a**) in A2780 and A2780cis cells and (**b**) in HCT-8 and HCT-8ox cells alone or upon co-incubation with Ezatiostat-HCl, either without or with 24 or 48 h pre-incubation with the inhibitor before platinum drug treatment (mean ± SEM, *n* = 3–7).

GSTP1 was discovered to be a binding partner of BODIPY-cisplatin in all four cell lines used (Figure 2, spot 3). We found that the GSTP1 inhibitor Ezatiostat-HCl (Figure 1, [33]) could be applied at a concentration of up to 10 µM, without harming the cells (EC_50_ values were 33.69 and 31.97 µM in A2780 and A2780cis, respectively, and 67.79 and 61.60 µM in HCT-8 and HCT-8ox cells, respectively). Surprisingly, no effect could be observed when combining either platinum drug with a non-toxic concentration of Ezatiostat-HCl (2 or 10 µM in the pre-incubation experiments) in all cell lines used. The result was the same, independent of the incubation scheme: the tumor cells were exposed to the drug and inhibitor simultaneously for 72 h, there was a pre-incubation period with the inhibitor over 48 h before the cells were subjected to the platinum drug for the remaining time, or pre-incubation over 24 h was followed by platinum drug exposure over 48 h (Figure 5 and Figure S4a). 

**Figure 6:**
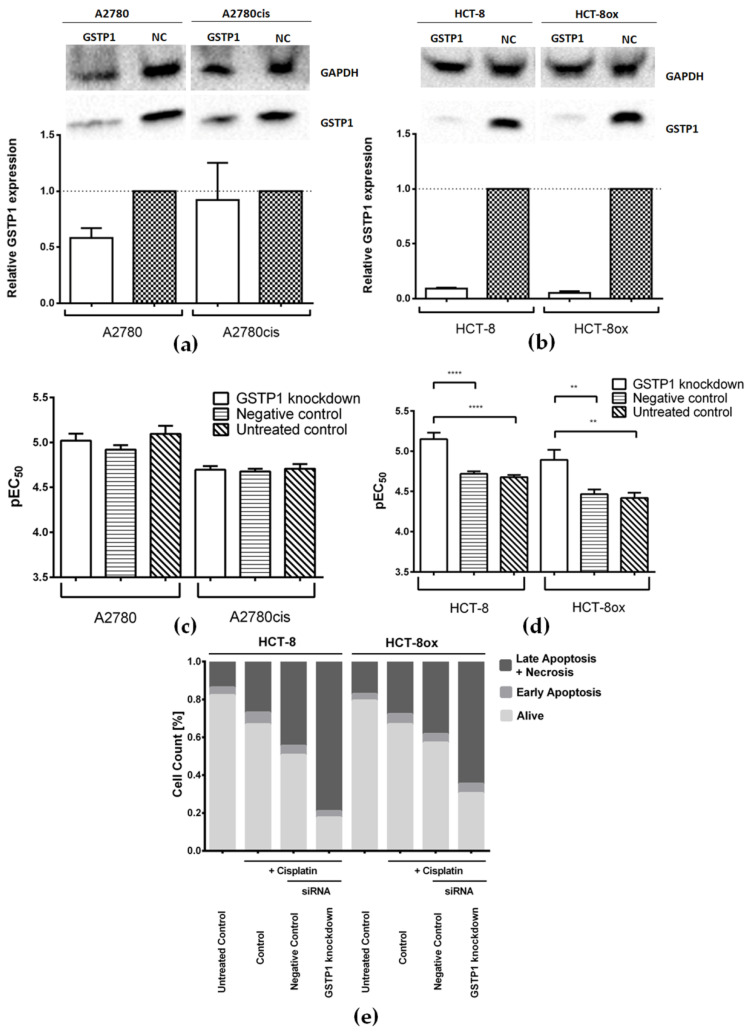
Representative Western Blots and densitometric quantification of protein expression after treatment with GSTP1-specific and negative control (NC) siRNA (**a**) in A2780 and A2780cis cells, and (**b**) in HCT-8 and HCT-8ox cells, where GAPDH served as a loading control (mean ± SEM, *n* = 3–5); cisplatin cytotoxicity (**c**) in A2780 and A2780cis cells, and (**d**) in HCT-8 and HCT-8ox cells after GSTP1 knockdown, prior treatment with negative control siRNA, or no pre-treatment (mean ± SEM, *n* = 4–7); (**e**) percentage of early apoptotic, late apoptotic, and necrotic, as well as alive, cells in HCT-8 and HCT-8ox cells after cisplatin treatment following GSTP1 knockdown, the negative knockdown control, or without knockdown. **, *p* < 0.01; ****, *p* < 0.0001.

While GSTP1 knockdown worked nearly perfectly in HCT-8 and HCT-8ox cells (decreased by 91% and 95%, respectively), the expression of GSTP1 could only be reduced by 42% in A2780 cells and by 8% in A2780cis cells (Figure 6a,b). Due to the low transfection efficiency in the ovarian carcinoma cell line pair, no difference in cisplatin sensitivity was detected after transfection (Figure 6c). On the contrary, Figure 6d shows an obvious and significant sensitization of both colorectal cancer cell lines to cisplatin after GSTP1 knockdown (EC_50_: HCT-8, 7.10 µM; HCT-8ox: 12.79 µM) compared to either the negative control (EC_50_: HCT-8, 19.10 µM; HCT-8ox, 34.28 µM) or cells without knockdown (EC_50_: HCT-8, 21.04 µM; HCT-8ox, 38.19 µM). As in the case of Grb2, all experiments concerning the HCT-8 and HCT-8ox cells were carried out with both cisplatin and oxaliplatin. When treated with oxaliplatin (Figure S4b), only HCT-8 cells showed a significant change in susceptibility to the drug compared to the negative knockdown control (EC_50_ decreased from 13.46 to 2.06 µM) or unmodified control (EC_50_ was reduced from 11.30 to 2.06 µM). The oxaliplatin-resistant HCT-8ox cell line showed a strong tendency to exhibit an elevated sensitivity compared to both controls (EC_50_ decreased from 26.49 µM in the negative control and from 27.80 µM in cells without knockdown to 6.34 µM); however, this difference was not statistically significant. 

These results were further validated by an apoptosis assay, which revealed a significant increase in late apoptosis and necrosis induced by oxaliplatin after GSTP1 knockdown in both the HCT-8 (+38.9% with respect to the negative control, *p* < 0.0001, and +53.2% compared to the unmodified control, *p* < 0.0001) and HCT-8ox cell line (+25.8% with respect to the negative control, *p* = 0.0021, and +40.0% compared to the unmodified control, *p* < 0.0001, Figures S5 and S2b). With cisplatin, the results were quite similar: HCT-8 cells showed a significant increase in late apoptosis and necrosis after the knockdown of GSTP1 compared to either the negative knockdown control (+34.5%, *p* = 0.0088) or cells without knockdown (+52.1%, *p* < 0.0001, Figure 6e and Figure S2c). In HCT-8ox cells, we could detect significantly elevated levels of cisplatin-induced late apoptosis and necrosis after GSTP1 knockdown when compared to the unmodified control (+36.8%, *p* = 0.0038), but not in comparison to negative knockdown controls, even though a strong tendency could be observed (+26.3%, *p* = 0.1014, Figure 6e and Figure S2c).

## 4. Discussion

As mentioned at the beginning, it is vitally important to determine new biomarkers and novel targets that will help to improve and personalize cancer chemotherapy. With this aim in mind, we combined two-dimensional gel electrophoresis and a fluorescently tagged cisplatin analog to identify cytosolic protein binding partners of the drug and to investigate their possible impact on cancer cell sensitivity. For this purpose, previous studies utilized a cisplatin analog bearing carboxyfluorescein diacetate [16,17], which has, in the meantime, been shown to be an inferior model of the parent drug in comparison to the fluorescent derivative featuring the BODIPY dye (BODIPY-cisplatin) employed here, especially concerning the cytotoxicity and imaging properties [21,34,35]. Even though BODIPY-cisplatin was less cytotoxic than the parent drug, cell lines with both intrinsic and acquired cisplatin resistance were cross-resistant to BODIPY-cisplatin, corresponding to the activity profile of cisplatin itself. These results suggest that BODIPY-cisplatin mimics the biological behavior of the parent drug in the cell lines studied.

After the treatment of cells with BODIPY-cisplatin, proteins in cytosolic fractions were separated utilizing two-dimensional gel electrophoresis. Via subsequent LC-MS analysis of spots positive for both fluorescence and protein staining, we were able to identify a total of 41 proteins as potential binding partners. Interestingly, ovarian cancer cells appeared to be more prone to intracellular protein–cisplatin interactions than the colorectal cancer cell lines employed. Moreover, sensitive A2780 ovarian cancer cells revealed more binding partners than their resistant counterpart, A2780cis. Defects in the cellular uptake of cisplatin likely account for this difference. Reduced cisplatin accumulation in A2780cis cells compared to the parent sensitive cell line is well-documented [36]. According to our earlier results, HCT-8 colorectal cancer cells accumulated significantly less cisplatin than A2780 ovarian cancer cells after 2 h incubation with 100 µM platinum drug (HCT-8: 10.47 ± 0.88 ng Pt/10^6^ cells, mean ± SEM, *n* = 6, [37]; A2780: 17.68 ± 0.23 ng Pt/10^6^ cells, mean ± SEM, *n* = 3, [38]; *p* < 0.001, unpaired *t*-test). Nevertheless, the fate of the drug in cytosol is also of great importance. For the ovarian carcinoma cell line pair used in this study, we have previously found that, whereas cisplatin uptake is reduced approximately 2-fold compared to the parent cell line, DNA platination is ca. five times lower [34]. Our results clearly show that some binding partners are shared, while the others differ between cell lines. In general, it must be taken into account that the identification of highly expressed proteins is more likely given the detection limit of colloidal Coomassie and the potential loss of lower expressed proteins throughout the identification process [39]. It should be noted that several of the identified proteins have already been reported as binding partners, e.g., vimentin; protein disulfide-isomerases PDIA1, PDIA3, and PDIA6; COP9 signalosome complex subunit 4; and elongation factor 1 alpha-1 [16,17,19,40]. However, only in rare cases have efforts been made to characterize their relevance for cell sensitivity [41,42,43,44,45]. To produce detailed studies, we selected one protein only identified in ovarian cancer cells, one found only in colorectal cancer cells, and one identified in all four cell lines. These are vimentin, Grb2, and GSTP1, respectively, as they have previously been described in connection with drug resistance [42,44,46,47,48,49]. Nevertheless, some other binding partners, particularly those only identified in colorectal cancer HCT-8 cells (such as ATP synthase subunit beta, mitochondrial, and phosphoglucomutase-2) or only found in resistant A2780cis cells (such as proliferating cell nuclear antigen, polyubiquitin-B, and COP9 signalosome complex subunit 4), are worth investigating further in light of their relevance for intrinsic and acquired cisplatin resistance, respectively. In the future, we will include other ovarian and colorectal cancer cell lines in our study, in order to obtain a more comprehensive picture of the role of binding partners in these two different types of resistance.

### 4.1. Vimentin

Vimentin is an interfilament protein involved in the epithelial–mesenchymal transition (EMT) process, which is a hallmark of metastatic tumor cells. In cancer progression, vimentin’s role as a scaffolding protein of the cytoskeleton and marker of EMT is just as important and its ability to interfere with and mediate certain signaling pathways has been extensively described [28,46,47,50,51]. Accordingly, vimentin appears to be upregulated in drug-resistant cancer cells of a great variety of entities. Its expression generally correlates with poor survival rates [28,52] and metastatic spread [53]. The study of Lazarova et al. focusing on colorectal cancer cells showed that vimentin is differentially expressed, depending on the metastatic state of the tumor, and proposed the hypothesis that the expression of vimentin in colonic neoplastic cells may correlate with the stage of neoplastic progression and that vimentin is the critical factor in colonic neoplastic progression [54]. Interestingly, we could not detect any vimentin expression in HCT-8 and HCT-8ox cells (Figure S6), which is in agreement with previous reports [55]. This explains why vimentin was not identified as a binding partner in HCT-8 and HCT-8ox cells. It remains unclear whether, and to what extent, vimentin expression correlates with intrinsic resistance to cisplatin in colorectal cancer. The inhibition of vimentin by FiVe1 significantly sensitized ovarian cancer cells to cisplatin. This synergistic interaction was observed at cisplatin concentrations comparable to those found in the plasma of patients [56]. The effect was more pronounced in the resistant cell line, although no differences in vimentin expression between A2780 and A2780cis cells were detected (Figure S6). In general, vimentin inhibitor FiVe1 acts through disorganizing and phosphorylating vimentin during the metaphase, leading to mitotic catastrophe [31]. Therefore, it appears that FiVe1 sensitizes cells to cisplatin through interference with vimentin’s function. Interestingly, paclitaxel, a drug that also leads to mitotic disruption, is commonly combined with platinum drugs in clinical practice with higher overall survival and progression-free survival in comparison to platinum treatment alone [57]. Our results show, for the first time, that the pharmacological inhibition of vimentin with FiVe1 may be a valuable approach for specifically enhancing sensitivity and tackling cisplatin resistance in ovarian cancer cells.

### 4.2. Grb2

Grb2 has repeatedly been investigated in the context of cancer; however, with contradictory results. While in some studies, the overexpression of Grb2 led to elevated tumor growth, invasiveness, and metastasis [29,49], Timsah et al. showed that the depletion of Grb2 resulted in Akt activation and tumor progression [43]. Nevertheless, we evaluated the effects of Grb2 inhibition and knockdown on cell sensitivity to find out whether the depletion of Grb2 as a binding partner of cisplatin could be favorable for the antitumor activity of platinum drugs. The toxicity of both Grb2 inhibitors chosen limited the concentration used in the experiments, and the IC_50_ of 5.7 mM (inhibitor A) and 320 µM (inhibitor B), as determined by Simister et al., could not be achieved [32]. Interestingly, we detected a tendency towards a lower sensitivity to cisplatin and a significant reduction in cytotoxicity in the case of oxaliplatin combined with inhibitor B. One potential explanation for this is that the inhibitors used may not have been effective enough or have had a different binding site than cisplatin. Another reason may be that survival signaling induced by the interference with Grb2 may have exceeded the effect expected from the decreased cisplatin binding in the cytosol. Grb2 knockdown was not sufficient to induce changes in susceptibility to the platinum drugs, especially in the resistant cell line. However, even with a more successful knockdown, we could expect similar results as those of pharmacological inhibition due to the survival signaling activated by the loss of Grb2.

### 4.3. GSTP1

GSTP1 is believed to play a significant role in the inactivation of drugs by conjugating them to glutathione with subsequent transportation out of the cells via, e.g., membrane-bound MRP efflux pumps [48,58,59]. The enzyme has been found to be overexpressed in a variety of solid tumors and has also been linked to malignant potential and poor outcomes [45,60]. Under normal conditions, GSTP1 is bound to c-Jun *N*-terminal kinase (JNK), preventing its phosphorylation and therefore inhibiting downstream signaling [30,33,48]. Upon oxidative stress, however, GSTP1 tends to dimerize and is hence unable to bind JNK, which then leads to the activation of pro-apoptotic pathways [30,33,48]. This mode of action is mimicked by the GSTP1 inhibitor Ezatiostat-HCl (also referred to as TLK199), which inhibits interactions between JNK and GSTP1 through binding to the latter [33]. The combination of the inhibitor with platinum drugs did not have any effect on the sensitivity of both ovarian and colorectal cancer cells, in contrast to the results of Li et al., which showed an enhanced sensitivity in a similar experimental setting. However, Li and colleagues used variable, partly very toxic, concentrations of Ezatiostat-HCl and also overexpressed GSTP1 prior to treatment with Ezatiostat-HCl [61]. This established a rather artificial setting and was therefore avoided in our study. Interestingly, we noted a significant sensitization of both intrinsically cisplatin-resistant colorectal cancer cell lines to this drug after GSTP1-siRNA transfection. Sensitization to oxaliplatin was only detected in the HCT-8 cells and the effect was much less pronounced. On the one hand, this may have been due to the lower reactivity of oxaliplatin towards nucleophiles [62]. On the other hand, several working groups have demonstrated that the conjugation to glutathione only plays a minor role in the development of cisplatin resistance and that intracellular signaling is much more significant [63,64]. Chen et al. found that the ROS/JNK pathway was mainly involved in the sensitization of mesothelioma cells to cisplatin upon GSTP1 knockdown, whereas the pathway seemed to be irrelevant for the effect of GSTP1 silencing on cell sensitivity to oxaliplatin [65]. In ovarian cancer cells, due to the relatively low knockdown efficiency, we did not observe any difference in the cisplatin cytotoxicity, in contrast to previous reports [66]. 

## 5. Conclusions

Using BODIPY-cisplatin followed by two-dimensional gel electrophoresis and mass spectrometry, we were able to identify different protein binding patterns in A2780, HCT-8, and their cisplatin- and oxaliplatin-resistant sublines, respectively. We further characterized the relevance of three of these proteins for the sensitivity of the cells mentioned above to platinum drugs using pharmacological inhibition and siRNA-mediated knockdown. Interestingly, GSTP1 knockdown sensitized intrinsically resistant colorectal cancer cells to cisplatin. This finding implies the possible involvement of GSTP1 in the intrinsic resistance of colorectal cancer cells to cisplatin and may offer a great opportunity to further elucidate the distinct mechanisms of action of cisplatin vs. oxaliplatin in this tumor entity. We also demonstrated that the inhibition of vimentin with the recently developed inhibitor FiVe1 led to a significant sensitization of ovarian cancer cells to cisplatin and to a reversal of resistance in the cisplatin-resistant cell line. These results warrant a further evaluation of vimentin as a target of antitumor therapy and FiVe1 in a possible combination with cisplatin. In future studies, we will characterize the relevance of other binding partners for platinum drug sensitivity in a broad range of ovarian and colorectal cancer cell lines.

## Figures and Tables

**Table 1:** Cytotoxicity (pEC_50_, mean ± SEM, *n* = 5–8) of cisplatin, oxaliplatin, BODIPY-cisplatin, and carboxyl-BODIPY in A2780, A2780cis, HCT-8, and HCT-8ox cells (the respective EC_50_ values are given in parentheses).

Compound	A2780	A2780cis	HCT-8	HCT-8ox
Cisplatin	5.932 ± 0.037 (1.17 µM)	5.312 ± 0.021 (4.88 µM)	5.259 ± 0.031 (5.51 µM)	5.037 ± 0.047 (9.18 µM)
Oxaliplatin	6.370 ± 0.093 (0.43 µM)	5.883 ± 0.085 (1.31 µM)	6.059 ± 0.027 (0.87 µM)	4.569 ± 0.060 (26.98 µM)
BODIPY-cisplatin	4.742 ± 0.034 (18.11 µM)	4.007 ± 0.002 (98.40 µM)	4.028 ± 0.052 (93.76 µM)	3.781 ± 0.028 (165.58 µM)
Carboxyl-BODIPY	<3.301 (>500 µM)	<3.301 (>500 µM)	<3.301 (>500 µM)	<3.301 (>500 µM)

## Supplementary Materials

The following figures and tables are available online at https://www.mdpi.com/2073-4409/9/6/1322/s1: Figure S1: Overlay of fluorescence image and protein staining (Coomassie) in representative gels after two-dimensional electrophoresis (pH 3–10) of each 20 µg cytosolic fraction of (a) A2780, (b) A2780cis, (c) HCT-8, and (d) HCT-8ox cells treated with BODIPY-cisplatin, Figure S2: Flow cytometry analyses of Annexin V-FITC/propidium iodide double staining (a) in A2780 and A2780cis cells after the co-incubation of cisplatin with FiVe1 (lower right quadrant) in comparison to treatment with the inhibitor (upper right quadrant) or cisplatin (lower left quadrant) alone and untreated cells (upper left quadrant); in HCT-8 and HCT-8ox cells after (b) oxaliplatin or (c) cisplatin treatment following GSTP1 knockdown (lower right quadrant), negative control siRNA treatment (lower left quadrant), or without knockdown (upper right quadrant) or untreated cells (upper left quadrant), Figure S3: Oxaliplatin cytotoxicity (a) in HCT-8 and HCT-8ox cells alone or upon co-incubation with either 1 µM inhibitor A or 50 µM inhibitor B (mean ± SEM, *n* = 5–6) and (b) in HCT-8 and HCT-8ox cells alone or upon co-incubation with 2 µM Ezatiostat-HCl, either without or with 24 or 48 h pre-incubation with the inhibitor before platinum drug treatment (mean ± SEM, *n* = 3–7), Figure S4: Oxaliplatin cytotoxicity in HCT-8 and HCT-8ox cells after (a) Grb2 knockdown and (b) GSTP1 knockdown, prior treatment with negative control siRNA, or no pre-treatment (mean ± SEM, *n* = 4–7), Figure S5: Percentage of early apoptotic, late apoptotic, and necrotic, as well as alive, cells in HCT-8 and HCT-8ox cells after oxaliplatin treatment following GSTP1 knockdown, negative knockdown control, without knockdown, Figure S6: Representative Western Blots of basal vimentin expression in A2780, A2780cis, HCT-8, and HCT-8ox cells. GAPDH served as a loading control; Table S1: Intracellular protein binding partners of BODIPY-cisplatin in the cell lines investigated.
