# Supplementary material for: Cisplatin Protein Binding Partners and Their Relevance for Platinum Drug Sensitivity
Source: Cells. 2020 May 26;9(6):1322. doi: 10.3390/cells9061322 (PMC7349790; doi:10.3390/cells9061322)
Supplement: Supplementary file 1 [file cells-09-01322-s001.pdf]

Cisplatin protein binding partners and their relevance for platinum drug sensitivity

Sophie Möltgen, Eleonora Piumatti, Giuseppe M. Massafra, Sabine Metzger, Ulrich Jaehde, Ganna V. Kalayda

Supplementary Information

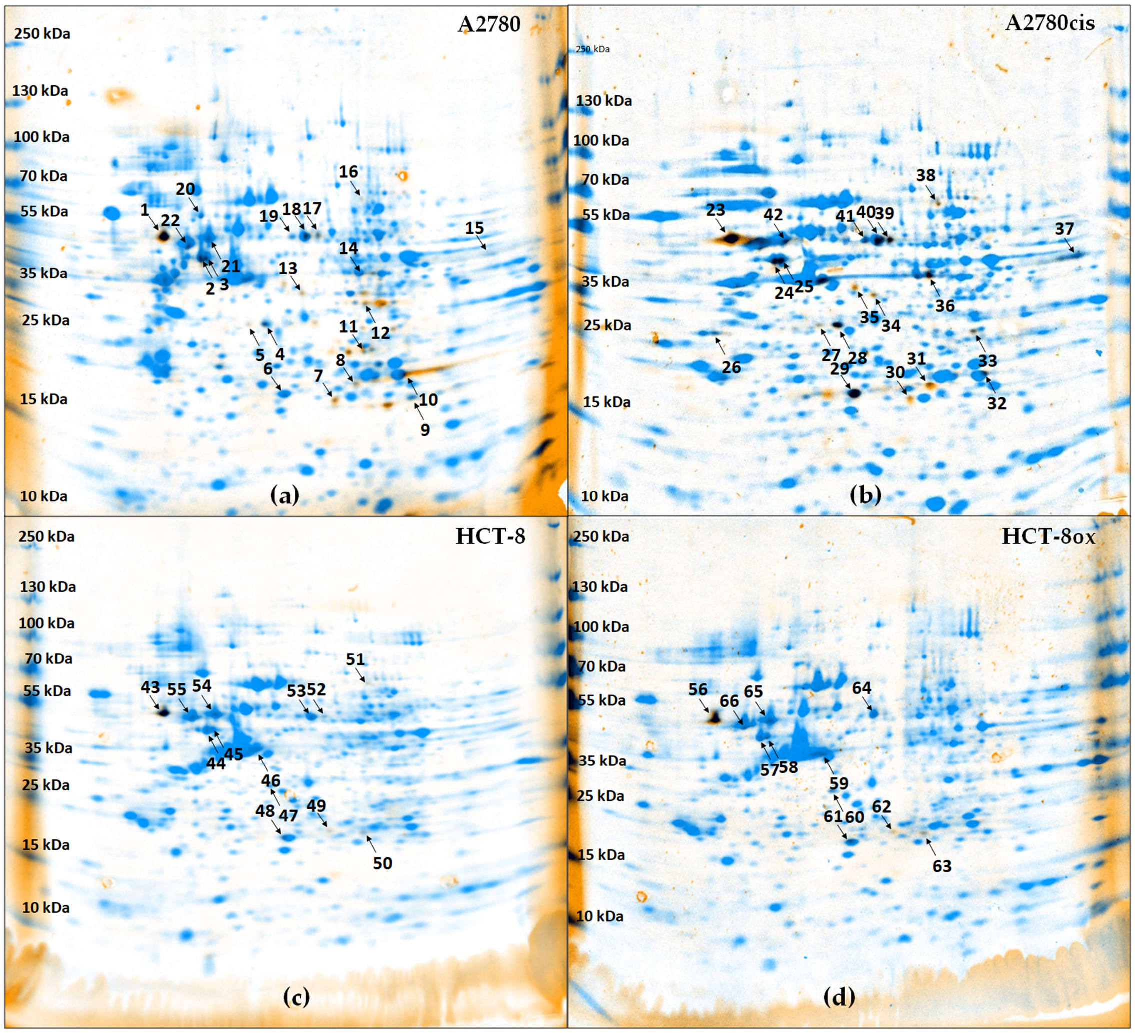

Figure S1. Overlay of fluorescence image and protein staining (Coomassie) in representative gels after two-dimensional electrophoresis (pH 3-10 NL) of each 20 µg cytosolic fractions of (**a**) A2780, (**b**) A2780cis, (**c**) HCT-8, and (**d**) HCT-8ox cells treated with BODIPY-cisplatin.

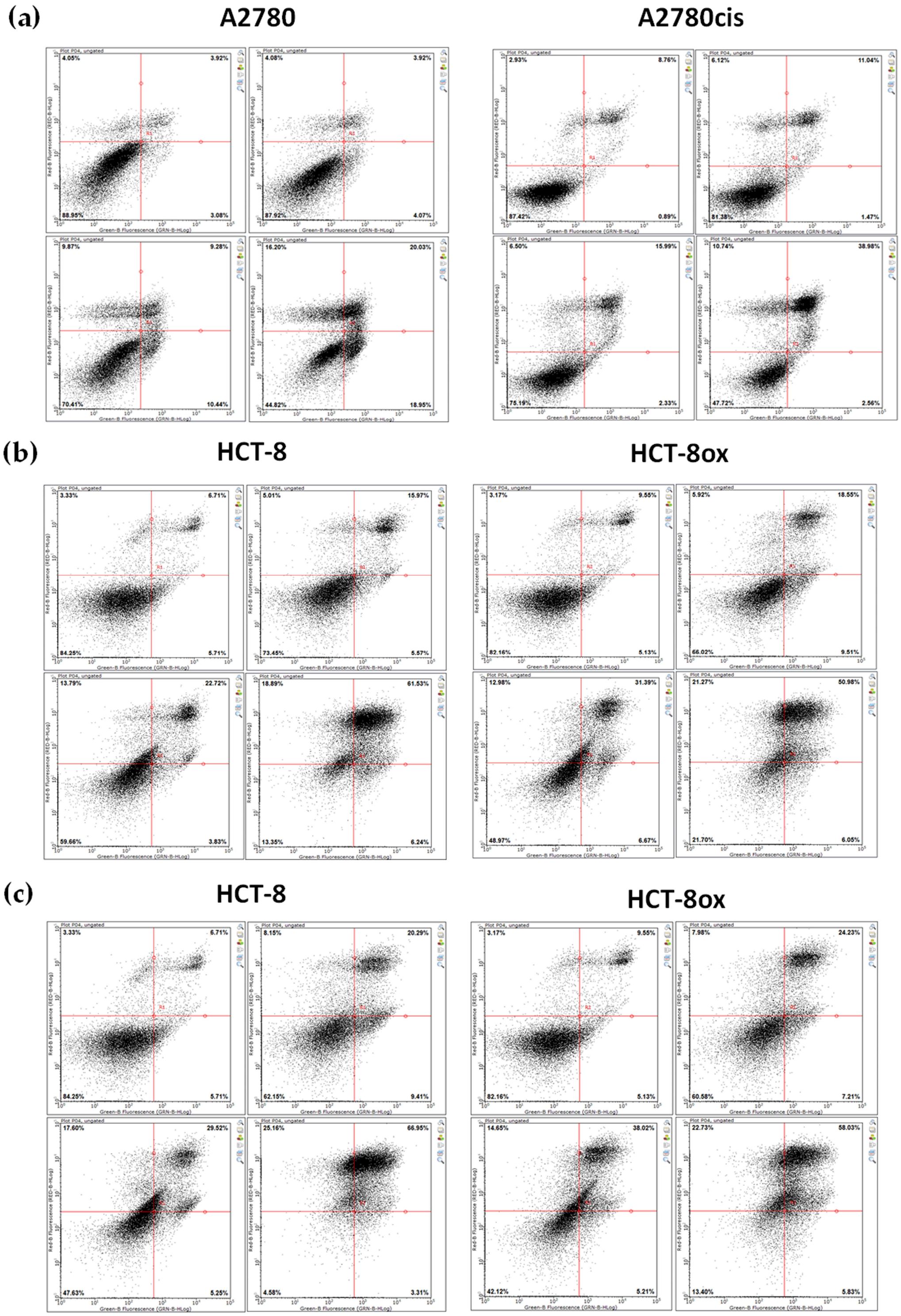

Figure S2. Flow cytometry analyses of Annexin V-FITC / propidium iodide double staining (**a**) in A2780 and A2780cis cells after co-incubation of cisplatin with FiVe1 (lower right quadrant) in comparison to the treatment with the inhibitor (upper right quadrant) or cisplatin (lower left quadrant) alone and untreated cells (upper left quadrant); in HCT-8 and HCT-8ox cells after (**b**) oxaliplatin or (**c**) cisplatin treatment following GSTP1 knockdown (lower right quadrant) or negative control siRNA treatment (lower left quadrant) or without knockdown (upper right quadrant) or untreated cells (upper left quadrant).

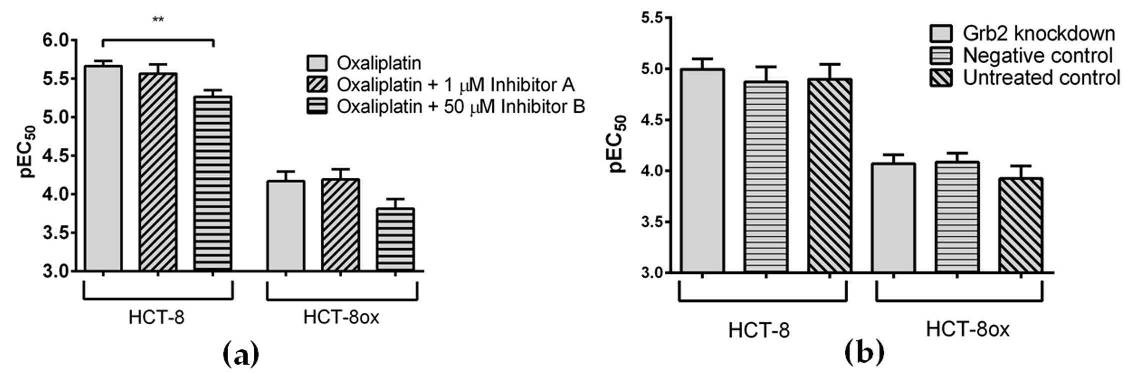

Figure S3. Oxaliplatin cytotoxicity (**a**) in HCT-8 and HCT-8ox cells alone or upon co-incubation with either 1 μM inhibitor A or 50 μM inhibitor B (mean ± SEM, n = 5-6) and (**b**) in HCT-8 and HCT-8ox cells after Grb2 knockdown, prior treatment with negative control siRNA or no pre-treatment (mean ± SEM, n = 4-6).

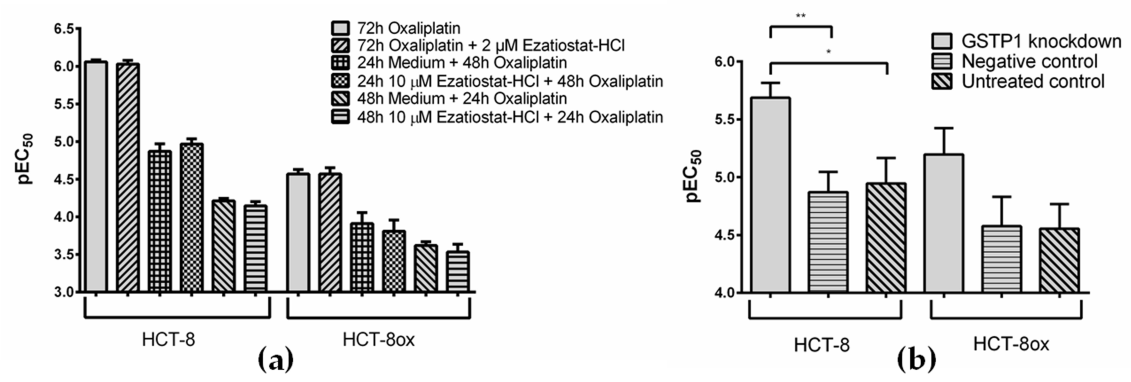

Figure S4. Oxaliplatin cytotoxicity (**a**) in HCT-8 and HCT-8ox cells alone or upon co-incubation with Ezatiostat-HCl either without or with 24h or 48h pre-incubation with the inhibitor before platinum drug treatment and (**b**) in HCT-8 and HCT-8ox cells after GSTP1 knockdown, prior treatment with negative control siRNA or no pre-treatment (mean ± SEM, n = 3-7).

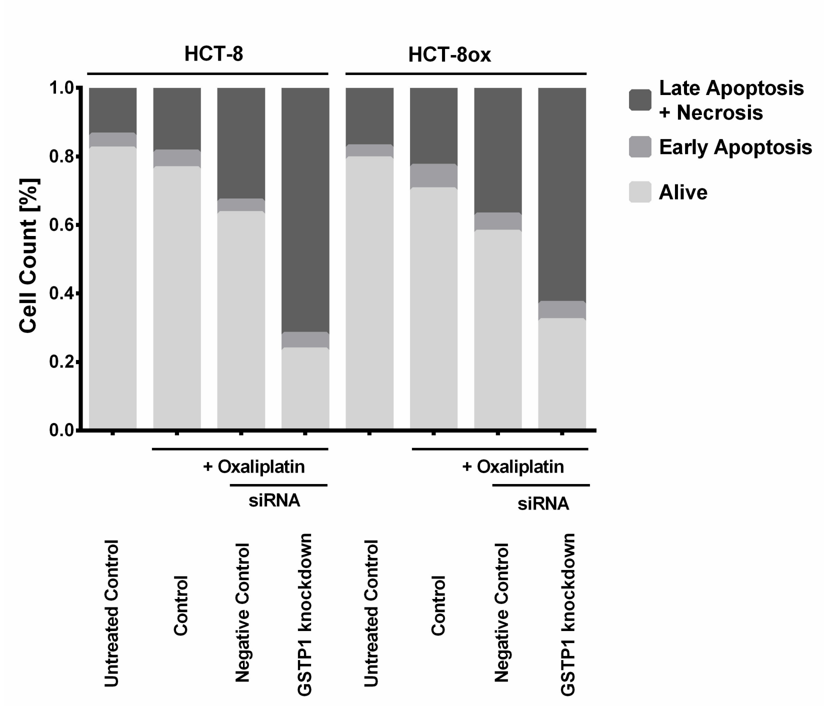

Figure S5. The percentage of early apoptotic, late apoptotic and necrotic as well as alive cells in HCT-8 and HCT-8ox cells after oxaliplatin treatment following GSTP1 knockdown or negative knockdown control or without knockdown.

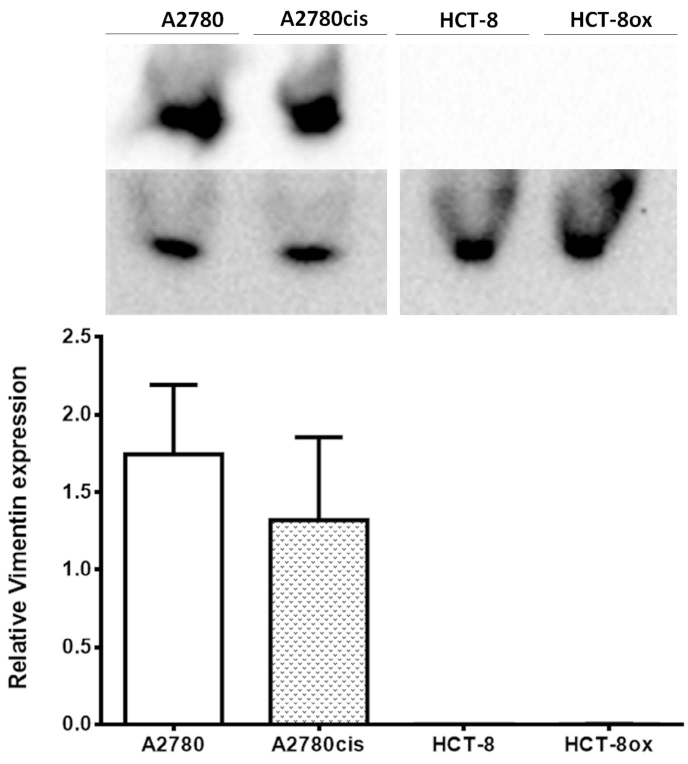

Figure S6. Representative Western Blot and densitometric quantification of basal vimentin expression in A2780, A2780cis, HCT-8 and HCT-8ox cells (mean ± SEM, n = 3). GAPDH served as a loading control.

Table S1. Intracellular protein binding partners of BODIPY-cisplatin in cell lines investigated.

Cell line	Spot #	Protein	Accession #	M_r (kDa)	pI	Sequence coverage (%)
**A2780**	1	Protein disulfide-isomerase A1	P07237	57.1	4.87	31
	2	Protein disulfide-isomerase A6	Q15084	48.1	5.08	22
	3	Protein disulfide-isomerase A6	Q15084	48.1	5.08	37
	4	F-actin-capping protein subunit alpha-1	P52907	32.9	5.69	30
	5	Heme oxygenase 2	P30519	36	5.41	29
	6	Glutathione-S-transferase P	P09211	23.3	5.64	16
	7	Protein/nucleic acid deglycase DJ-1	Q99497	19.9	6.79	24
	8	Proteasome subunit beta type-3	P49720	22.9	6.55	35
	9	Flavin reductase (NADPH)	P30043	22.1	7.65	34
	10	GTP-binding nuclear protein Ran	P62826	24.4	7.49	24
	11	Proteasome subunit alpha type 1	P25786	29.5	6.61	26
	12	Apolipoprotein L2	Q9BQE5	37.1	6.74	16
		Transaldolase	P37837	37.5	6.81	29
	13	L-lactate dehydrogenase B chain	P07195	36.6	6.05	32
	14	26S proteasome non-ATPase regulatory subunit 11	O00231	47.4	6.48	55
		Adenosylhomocysteinase	P23526	47.7	6.34	35
	15	Elongation factor 1-alpha 1	P68104	50.1	9.01	19
	16	Succinate dehydrogenase [ubiquinone] flavoprotein subunit, mitochondrial	P31040	72.6	7.39	16
	17	Protein disulfide-isomerase A3	P30101	56.7	6.35	19
	18	Protein disulfide-isomerase A3	P30101	56.7	6.35	54
	19	Protein disulfide-isomerase A3	P30101	56.7	6.35	36
	20	Serine/threonine-protein phosphatase 2A 65 kDa regulatory subunit A alpha isoform	P30153	65.3	5.11	28
	21	Vimentin	P08670	53.6	5.12	63
		Nucleobindin-1	Q02818	53.8	5.25	58
	22	Vimentin	P08670	53.6	5.12	76

Cell line	Spot #	Protein	Accession #	M_r (kDa)	pI	Sequence coverage (%)
**A2780cis**	23	Protein disulfide-isomerase A1	P07237	57.1	4.87	49
	24	Protein disulfide-isomerase A6	Q15084	48.1	5.08	51
		Nucleobindin-2	P80303	50.2	5.12	64
		26S proteasome regulatory subunit 6B	P43686	47.3	5.21	46
	25	Protein disulfide-isomerase A6	Q15084	48.1	5.08	34
	26	Proliferating cell nuclear antigen	P12004	28.8	4.69	56
	27	Heme oxygenase 2	P30519	36	5.41	22
	28	F-actin-capping protein subunit alpha-1	P52907	32.9	5.69	15
	29	Glutathione S-transferase P	P09211	23.3	5.64	71
	30	Protein/nucleic acid deglycase DJ-1	Q99497	19.9	6.79	25
	31	Heat shock protein beta-1	P04792	22.8	6.4	49
		Proteasome subunit beta type-3	P49720	22.9	6.55	21
	32	GTP-binding nuclear protein Ran	P62826	24.4	7.49	21
	33	Polyubiquitin-B	P0CG47	25.7	7.43	56
	34	L-lactate dehydrogenase B chain	P07195	36.6	6.05	32
	35	COP9 signalosome complex subunit 4	Q9BT78	46.2	5.83	15
	36	Adenosylhomocysteinase	P23526	47.7	6.34	36
		26S proteasome non-ATPase regulatory subunit 11	O00231	47.4	6.48	25
	37	Elongation factor 1-alpha 1	P68104	50.1	9.01	17
	38	Succinate dehydrogenase [ubiquinone] flavoprotein subunit, mitochondrial	P31040	72.6	7.39	25
	39	Protein disulfide-isomerase A3	P30101	56.7	6.35	24
	40	Protein disulfide-isomerase A3	P30101	56.7	6.35	69
	41	Protein disulfide-isomerase A3	P30101	56.7	6.35	41
	42	Nucleobindin-1	Q02818	53.8	5.25	25
		Vimentin	P08670	53.6	5.12	19

Cell line	Spot #	Protein	Accession #	M_r (kDa)	pI	Sequence coverage (%)
**HCT-8**	43	Protein disulfide-isomerase A1	P07237	57.1	4.87	53
	44	Protein disulfide-isomerase A6	Q15084	48.1	5.08	40
		Nucleobindin-2	P80303	50.2	5.12	54
		26S proteasome regulatory subunit 6B	P43686	47.3	5.21	41
	45	Protein disulfide-isomerase A6	Q15084	48.1	5.08	36
	46	Eukaryotic initiation factor 4A-I	P60842	46.1	5.48	19
	47	F-actin-capping protein subunit alpha-1	P52907	32.9	5.69	36
	48	Glutathione S-transferase P	P09211	23.3	5.64	73
	49	Growth factor receptor-bound protein 2	P62993	25.2	6.32	28
	50	Proteasome subunit beta type-3	P49720	22.9	6.55	47
	51	Phosphoglucomutase-2	Q96G03	68.2	6.73	21
	52	Protein disulfide-isomerase A3	P30101	56.7	6.35	40
	53	Protein disulfide-isomerase A3	P30101	56.7	6.35	65
	54	ATP synthase subunit beta, mitochondrial	P06576	56.5	5.4	41
		Nucleobindin-1	Q02818	53.8	5.25	39
	55	ATP-Synthase subunit beta, mitochondrial	P06576	56.5	5.4	25
**HCT-8ox**	56	Protein disulfide-isomerase A1	P07237	57.1	4.87	37
	57	Protein disulfide-isomerase A6	Q15084	48.1	5.08	26
	58	Protein disulfide-isomerase A6	Q15084	48.1	5.08	41
	59	Eukaryotic initiation factor 4A-I	P60842	46.1	5.48	33
	60	F-actin-capping protein subunit alpha-1	P52907	32.9	5.69	49
	61	Glutathione S-transferase P	P09211	23.3	5.64	70
	62	Growth factor receptor-bound protein 2	P62993	25.2	6.32	45
	63	Proteasome subunit beta type-3	P49720	22.9	6.55	21
	64	Protein disulfide-isomerase A3	P30101	56.7	6.35	57
	65	ATP synthase subunit beta, mitochondrial	P06576	56.5	5.4	25
		Nucleobindin-1	Q02818	53.8	5.25	24
	66	ATP-Synthase subunit beta, mitochondrial	P06576	56.5	5.4	34
